# Global Genome Demethylation Causes Transcription-Associated DNA Double Strand Breaks in HPV-Associated Head and Neck Cancer Cells

**DOI:** 10.3390/cancers13010021

**Published:** 2020-12-23

**Authors:** Michael Hajek, Asel Biktasova, Andrew Sewell, Cyril Gary, Paul Cantalupo, Karen S. Anderson, Wendell G. Yarbrough, Natalia Issaeva

**Affiliations:** 1 Department of Surgery, Otolaryngology, Yale University, New Haven, CT 06519-1369, USA; michael.hajek@yale.edu (M.H.); abiktasova@gmail.com (A.B.); sewellab@me.com (A.S.); cyril.gary@gmail.com (C.G.); 2 Department of Biological Sciences, University of Pittsburgh, Pittsburgh, PA 15260, USA; pcantalupo@gmail.com; 3 Department of Pharmacology, Yale University School of Medicine, New Haven, CT 06519-1369, USA; karen.anderson@yale.edu; 4 Department of Molecular Biophysics and Biochemistry, Yale University School of Medicine, New Haven, CT 06519-1369, USA; 5 Department of Otolaryngology/Head and Neck Surgery, The University of North Carolina, Chapel Hill, NC 27599-7070, USA; 6 Department of Pathology and Lab Medicine, The University of North Carolina, Chapel Hill, NC 27599, USA; 7 Lineberger Comprehensive Cancer Center, The University of North Carolina, Chapel Hill, NC 27599, USA

**Keywords:** head and neck cancer, HPV, demethylation, DNA double strand breaks, transcription, replication

## Abstract

**Simple Summary:**

High levels of global genome methylation in HPV-associated head and neck cancer prompted us to explore demethylation as a potential treatment by determining mechanisms of its toxicity in HPV-positive head and neck cancer cells. Previously, we reported that demethylating drug 5-azaC stabilizes p53 and reduces the expression of HPV genes and matrix metalloproteinases in HPV+ head and neck cancer cells and tumors from patients enrolled in a 5-azaC window clinical trial. Here, we extended our understanding of toxicity caused by global demethylation in HPV-associated head and neck cancer cells by finding that 5-azaC treatment results in formation of DNA double strand breaks that depend on transcription and replication.

**Abstract:**

High levels of DNA methylation at CpG loci are associated with transcriptional repression of tumor suppressor genes and dysregulation of DNA repair genes. Human papilloma virus (HPV)-associated head and neck squamous cell carcinomas (HNSCC) have high levels of DNA methylation and methylation has been associated with dampening of an innate immune response in virally infected cells. We have been exploring demethylation as a potential treatment in HPV+ HNSCC and recently reported results of a window clinical trial showing that HNSCCs are particularly sensitive to demethylating agent 5-azacytidine (5-aza). Mechanistically, sensitivity is partially due to downregulation of HPV genes expression and restoration of tumor suppressors p53 and Rb. Here, for the first time, we show that 5-azaC treatment of HPV+ HNSCC induces replication and transcription-associated DNA double strand breaks (DSBs) that occur preferentially at demethylated genomic DNA. Blocking replication or transcription prevented formation of DNA DSBs and reduced sensitivity of HPV-positive head and neck cancer cells to 5-azaC, demonstrating that both replication and active transcription are required for formation of DSBs associated with 5-azaC.

## 1. Introduction

Head and neck squamous cell carcinoma (HNSCC) is the sixth most common cancer in the world and is associated with poor prognosis in advanced cases [1,2]. Decreased tobacco consumption has paralleled falling rates of tobacco-associated HNSCC in the United States; however, a subset of HNSCC arising in the oropharynx (OPSCC) and caused by the human papillomavirus (HPV) has been rapidly increasing. By 2012, the incidence of HPV-associated HNSCC in the U.S. was higher than any HPV-associated cancer including uterine cervical cancer [3,4,5]. Advanced HNSCC are treated with combinations of primary surgical resection, cervical lymphadenectomy, radiation, or radiation given with platin drugs, depending on institutional preference and tumor characteristics. Side effects of concurrent radiation and chemotherapy are severe and may be lifelong: swallowing and speech dysfunction, accelerated arteriosclerosis of neck vessels, neck muscle fibrosis, xerostomia, accelerated dental decay, and lymphedema. Extended survival analysis of trials comparing radiation and chemotherapy regimens suggests that side effects of concomitant chemotherapy and radiation may decrease overall survival in the absence of recurrent tumor [6]. Despite aggressive and morbid therapy, up to 25% of patients with HPV-associated (HPV+) head and neck tumors suffer recurrent or metastatic disease, for which treatment options are limited. Available HPV vaccines hold tremendous promise for prevention of HPV+ head and neck cancer; however, given the latency between infection and the development of HPV+ HNSCC, estimates suggest that the HPV vaccine will not decrease HNSCC prevalence until 2060 [7]. Thus, insight into vulnerabilities and development of less morbid, yet effective treatments for primary and recurrent HPV+ HNSCC are needed.

As a platform to identify inherent vulnerabilities for discovery of new therapies to treat the growing population of patients with HPV+ HNSCC, we characterized biologic and molecular differences between HPV+ and HPV- HNSCC. In addition to differences in mutation patterns [8,9], gene expression, and protein abundance profiles [10,11,12,13], we described differences in genome methylation [14,15]. Upon activation and metabolic transformation into active nucleotides, the cytosine analogs 5-azacytidine (5-azaC) and decitabine are incorporated into cellular DNA resulting in global DNA demethylation [16]. These drugs are used to treat hematologic malignancies and premalignancies with their primary cytotoxic effects thought to be mediated through promoter demethylation leading to expression of tumor suppressors [17]. 5-azaC and decitabine also stimulate the DNA damage response through reactivation of DNA damage response genes that are recruited to DNA bound by trapped DNA methyltransferases [18,19,20]. Due to the hypermethylation associated with HPV positivity, we explored demethylation as a novel targeted treatment for HPV+ HNSCC [21]. Results from the window trial of 5-azaC in HPV+ HNSCC were promising, with expression of all HPV genes, including E6 and E7 oncogenes, being downregulated in tumors after 5-azaC treatment. In HPV+ HNSCC, downregulation of E6 was associated with increased p53 levels and activity contributing to tumor cell death. Here, we probed underlying molecular mechanisms of demethylation associated cytotoxicity in HPV+ HNSCC, finding that demethylation of HPV+ HNSCC resulted in pathologic DNA double strand breaks (DSBs). DSBs following demethylation depended on replication, and interestingly, demethylation was connected to DSBs through their dependence on transcription that was markedly increased after demethylation and because breaks occurred preferentially at demethylated DNA.

## 2. Results

### 2.1. 5-Azacytidine Causes DNA Double Strand Breaks in HPV-Associated Head and Neck Cancer Cells

**Figure 1:**
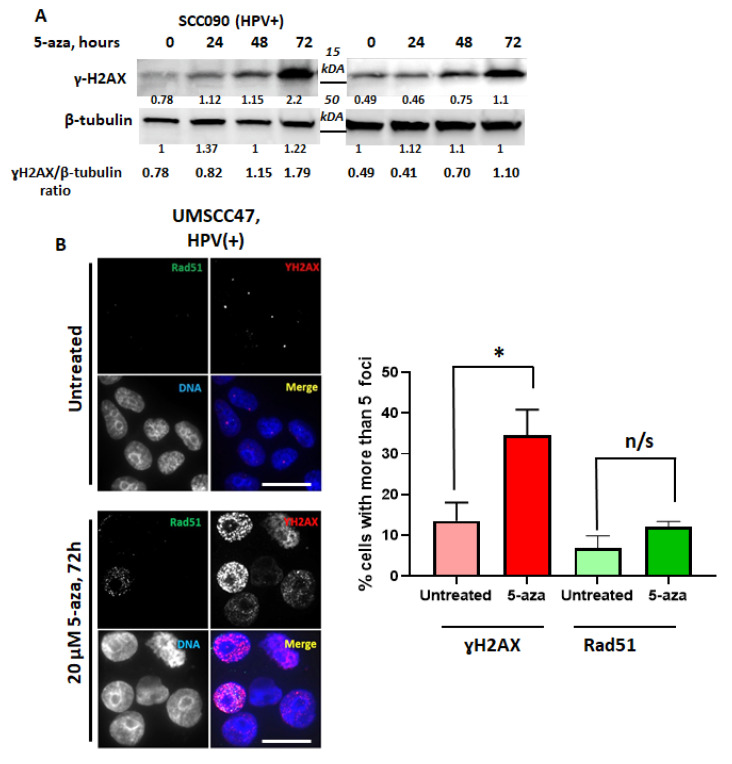
5-azacytidine induces DNA damage in HPV-positive head and neck cancer cells but does not induce Rad51 foci formation. (**A**) HPV+ cell lines SCC090 and UMSCC47 were treated with 20 µM 5-azaC for 24, 48, and 72 h then immunoblotted with antibodies against ɣH2AX and β-tubulin as a control. (**B**) HPV+ cells, UMSCC47, were treated with 20 µM of 5azaC for 72 h and subsequently fixed and immunostained with ɣH2AX and Rad51 antibodies; representative images (left) and quantification (right) are shown. Scale bars, 10 μm. Values indicate the mean ± SD with *n* ≥ 50 cells in 2 biological replicates. Student’s *t* test was performed to test significance. * *p*-value < 0.05; n/s (not significant) indicates *p*-value > 0.05.

We previously reported that HPV+ HNSCCs are significantly more sensitive to 5-azaC than HPV- HNSCC, and this sensitivity was partially due to downregulation of HPV gene expression, marked stabilization and activation of p53, and induction of apoptosis [21]. Since 5-azaC and decitabine cause replication-associated DNA damage, we questioned whether DNA damage may be an additional factor contributing to toxicity. Repair of 5-azaC associated DNA damage requires Fanconi anemia (FA)-dependent homologous recombination (HR) [20], and enhanced sensitivity of HPV+ tumors may be due to partially defective HR repair in HPV+ HNSCC [22,23,24]. To begin exploring if DNA damage and DNA damage response contribute to 5-azaC induced toxicity in HPV+ HNSCC, DNA damage was monitored daily after 5-azaC treatment. As indicated by the increased levels of ɣH2AX, 5-azaC induced DNA damage in HPV+ (SCC090 and UMSCC47) cell lines [20,25] (Figure 1A). Rad51 is required for HR and colocalizes with ɣH2AX at stalled replication forks caused by 5-azaC or decitabine [20,25,26]. To determine if HR was involved in the repair of lesions after demethylation, ɣH2AX and Rad51 immunofluorescent staining was performed. Confirming DNA damage indicated by immunoblots (Figure 1A), 5-azaC treatment of HPV+ HNSCC cells increased ɣH2AX foci, but RAD51 was not induced and poorly colocalized with ɣH2AX in HPV+ UMSCC47 (Figure 1B). These data are consistent with reports describing defective homologous recombination in HPV+ cells [22,23,24].

**Figure 2:**
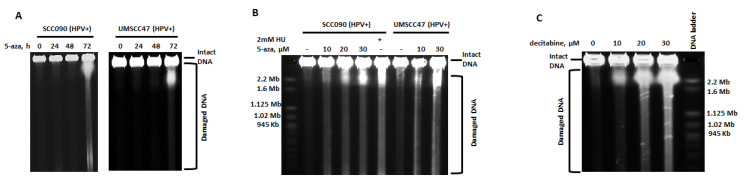
5-azacytidine and decitabine treatment cause DNA double strand breaks in HPV+ HNSCC cells. (**A**) Pulsed-field gel electrophoresis (PFGE) depicting HPV+ SCC090 and HPV+ UMSCC47 cells after treatment with 30 µM 5-azaC for 0, 24, 48, or 72 h. (**B**) PFGE depicting HPV+ cell lines SCC090 and UMSCC47 after 72 h of treatment with indicated doses of 5-azaC. Treatment of cells for 72 h with 2 mM hydroxyurea (HU) was used as a positive control. (**C**) PFGE depicting HPV+ cell line UMSCC47 after 72 h of treatment with indicated doses of decitabine.

To confirm and further characterize the nature of 5-azaC-induced DNA damage, pulsed-field gel electrophoresis (PFGE) was employed. PFGE uses alternating electrical current to separate large genomic DNA fragments created by double strand breaks, but not by other types of DNA damage, including deamination, depurination, oxidation, alkylation, or single strand breaks [27,28,29]. DNA DSBs were detected 72 h after 5-azaC treatment in all HPV+ HNSCC cell lines tested (SCC090, UMSCC47, and UMSCC104) (Figure 2A and Figure S1) and were observed at concentrations ranging from 1 to 30 µM and increased in a dose-dependent manner (Figure 2B and Figure S2). In addition to cell lines, primary early passage cells (passage 3) derived from an HPV+ tonsil squamous cell carcinoma also revealed DSBs following 72 h of 5-azaC treatment (Figure S1).

To determine if DSBs in response to 5-azaC were specific to HPV+ HNSCC, we assayed a panel of HPV-negative normal and cancer cells, including osteosarcoma cells U2OS, colon cancer cells HCT116, human kidney epithelial cells 293T, and head and HPV-negative head and neck cancer cells SCC35 (Figure S3). 5-azaC at concentrations up to 30 µM did not induce DNA DSBs in any of the tested cell lines. As expected, hydroxyurea (HU) induced formation of DSBs in SCC35 cells (Figure S3).

5-azaC incorporation into DNA after metabolic conversion is required for DNA demethylation, but 5-azaC is also incorporated into RNA. To determine if 5-azaC incorporation into RNA is required for DSBs formation, cells were treated with the 5-azaC analogue, decitabine (5-azaC-2′-deoxycytidine), which is only incorporated into DNA [16,30]. As observed with 5-azaC, increasing doses of decitabine also induced DSBs in a concentration-dependent manner (Figure 2C). These data suggest that incorporation of 5-azaC into DNA induces DSBs formation in HPV+ head and neck cancer cells and that DSBs are not cell line specific or dependent on the immortalization of cell lines.

**Figure 3:**
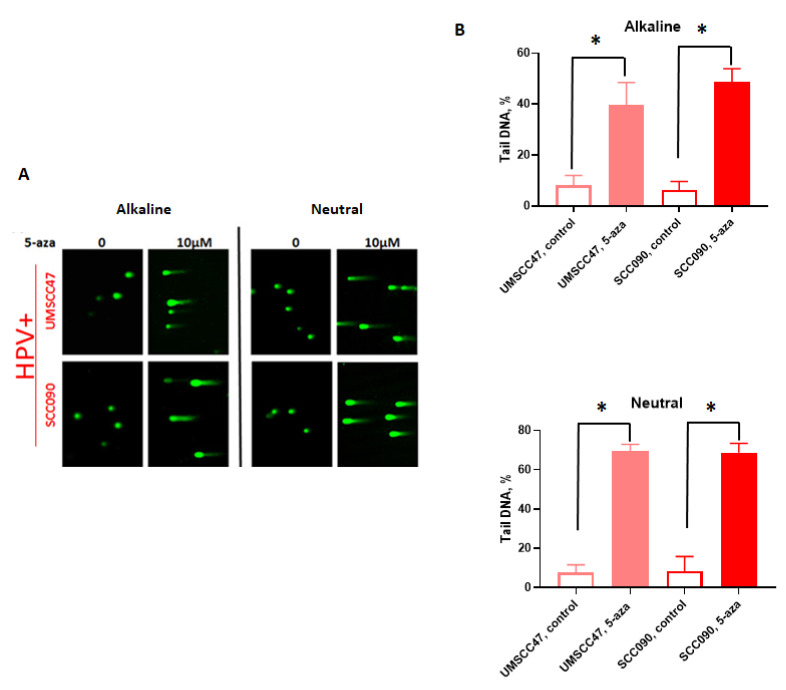
5-azacytidine induces DNA double strand breaks in HPV+ HNSCC cells. Representative images (20x) (**A**) and quantification of tail DNA (**B**) of Comet assays performed with control or 5-azaC treated HPV-positive HNSCC cells (UMSCC47 and SCC090). Student’s *t* test was performed to test significance. * *p*-value < 0.05.

To confirm the type of DNA damage after 5-azaC treatment of HPV+ HNSCC, cells (UMSCC47 and SCC090) were analyzed using the comet assay in which DSBs result in “tails” in both alkaline and neutral conditions, whereas single strand breaks (SSBs) cause tails only in alkaline conditions. Following 5-azaC treatment, tails were detected in both alkaline and neutral assay conditions (Figure 3A,B). These results are consistent with PFGE data and confirm that treatment of HPV+ HNSCC with 5-azaC induced DSBs. Representative photomicrographs and measurement of tail DNA content from at least 50 cells each are shown (Figure 3A,B).

### 2.2. Dysfunctional Homologous Recombination Increases Sensitivity to 5-azaC

**Figure 4:**
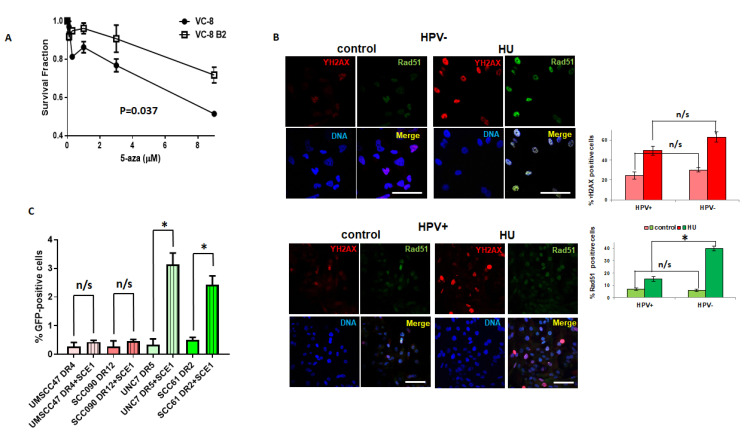
Homologous recombination deficient cells are sensitive to 5-azaC and HPV-positive head and neck cancer cells are deficient in a homologous recombination repair. (**A**) Clonogenic survival of Chinese hamster ovary cells lacking BRCA2 (VC-8) and cells with restored BRCA2 (VC-8 B2) after treatment with increasing doses of 5-azaC. Standard deviation calculated from 2 independent experiments. Student’s *t* test was performed to test significance. (**B**) Representative immunofluorescent images (**left**) and quantification (**right**) of ɣH2AX and Rad51 foci formation in HPV-negative (**top**) and HPV-positive (**bottom**) HNSCC cells after HU treatment. Scale bars, 50 μm. Values indicate the mean ± SD with *n* ≥ 50 cells in 2 biological replicates. Student’s *t* test was performed to test significance. * indicates *p*-value < 0.05; n/s (not significant) indicates *p*-value > 0.05. (**C**) Homologous recombination reporter results in HPV-positive (UMCSS47 and SCC090) and HPV-negative (UNC7 and SCC61) HNSCC cells stably expressing pDRGFP and transfected or not with I-SCE1. Values indicate the mean ± SD from 2 biological replicates. Student’s *t* test was performed to test significance. * indicates *p*-value < 0.05; n/s (not significant) indicates *p*-value > 0.05.

Compared to HPV-negative HNSCC, HPV+ HNSCC are more sensitive to DNA damaging agents, and reports indicate that homologous recombination (HR) is impaired in HPV+ HNSCC [22,23]. To determine if impaired double strand break repair sensitizes cells to 5-azaC, HR-deficient Chinese hamster ovary (VC8) cells that lack functional BRCA2 and VC8 cells with reconstituted BRCA2 (labeled as VC8 B2) [31] were analyzed for clonogenic survival following 5-azaC treatment. HR-deficient VC8 cells were significantly more sensitive to 5-azaC than isogenic HR competent cells (Figure 4A).

Previous studies indicate that HPV+ cells do not recruit BRCA2 or Rad51 to radiation-induced DNA damage foci, suggesting these cells harbor HR defect and agreeing with our data that showed absence of Rad51 colocalization with ɣH2AX after 5-azaC treatment (Figure 1B). Here, we expanded studies by analysis of HR components at foci of DNA damage in an additional HPV+ cell line and using hydroxyurea that stalls and collapses replication forks, a type of S-phase DNA damage for which HR is the preferred method of repair. We used HPV-negative HNSCC cells as control and compared foci after HU treatment with HPV+ HNSCC. Incubation of cells with HU caused DNA damage in both HPV+ and HPV-negative HNSCC cells as indicated by the appearance of ɣH2AX foci (Figure 4B); however homologous recombination, as marked by colocalized Rad51 foci, was activated only in HPV-negative cells (Figure 4B).

To more directly assay HR proficiency and compare HPV+ to HPV-negative cells, we performed an HR-GFP reporter assay in HPV-positive (UMSCC47 and SCC090) and negative (UNC7 and SCC61) HNSCC cells engineered to stably express pDRGFP [32]. Transfection of the I-SceI endonuclease induced green fluorescence in both HPV-negative cell lines suggesting repair by HR. On the other hand, an increase in GFP expression was not observed in either of the HPV+ cell lines (Figure 4C) supporting that these cells have a defect in HR.

Together, these data confirm HR defects in HPV+ HNSCC and suggest that sensitivity to 5-azaC is enhanced by HR deficiency.

### 2.3. Transcription and Replication are Required for 5-Azycytidine Induced DNA Double Strand Breaks

**Figure 5:**
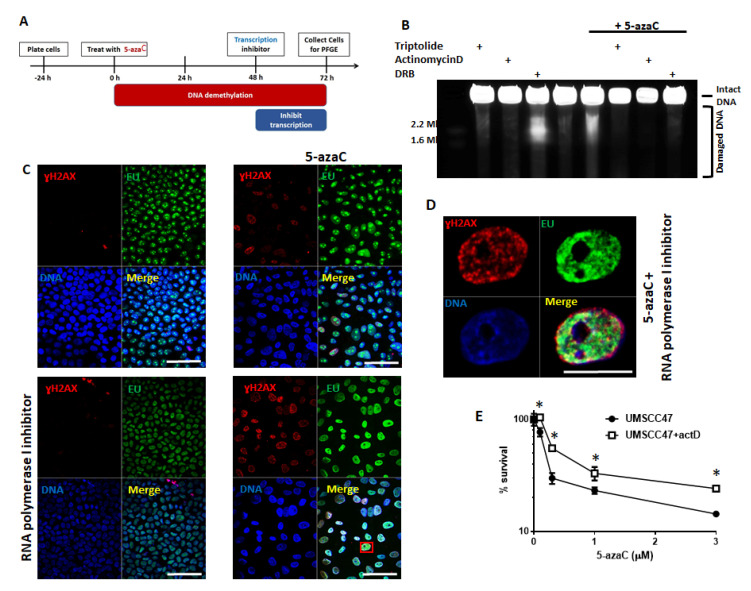
5-azaC induced DNA double strand breaks in HPV+ HNSCC depend on active transcription. (**A**) Schema of 72 h 5-azaC treatment with transcriptional inhibition during the last 24 h before analysis. (**B**) PFGE depicting HPV+ UMSCC47 cells after 72 h of 20 µM 5-azaC and addition of 1 µM triptolide, 1.25 µg/mL actinomycin D, or 10 µM DRB for the last 24 h. (**C** and **D**) Coimmunostaining of EU (active transcription) and ɣH2AX in HPV-positive UMCC47 cells treated or not with 10 µM of 5-azaC in the presence or absence of RNA polymerase I inhibitor elipticine. Scale bars are 50 (**C**) and 10 (**D**) µm. (**E**) Clonogenic survival of UMSCC47 cells either treated or not with 1 ng/mL actinomycin D 48 h after initiation of increasing doses of 5-azaC treatment (log scale). Standard deviations calculated from two independent experiments. Student’s *t* test was performed to test significance. * indicates *p*-value < 0.05.

Demethylating agents, such as 5-azaC, increase global transcription and aberrant transcription can lead to DNA DSBs and genomic instability [33,34]. To determine if transcription is required for DSBs formation in HPV+ HNSCC in response to 5-azaC, we inhibited various steps in transcription using: (1) triptolide, an RNA polymerase inhibitor [35], (2) actinomycin D, an RNA elongation inhibitor [36], and (3) dichloro-beta-D-ribofuranosylbenzimidazole (DRB), an inhibitor of RNA polymerase II. Transcription inhibition is toxic to cancer cells causing both cell cycle arrest and apoptosis [37,38,39]. Treatment with 5-azaC for 48 h before inhibition of transcription was chosen to allow its incorporation into DNA, but also since no DSBs were detected before 48 h of 5-azaC treatment (Figure 2A; treatment schema in Figure 5A). Transcription inhibition by any of the agents used markedly reduced or completely inhibited DSBs formation following 5-azaC treatment (Figure 5B, last 3 lanes). DRB treatment in the absence of 5-azaC causes DNA double strand breaks [40], but interestingly, DSBs induced by DRB in the absence of 5-azaC were prevented in cells treated with 5-azaC (compare lane 3 and lane 7, Figure 5B). These data reveal that transcription is required for 5-azaC to induce DSBs in HPV+ HNSCC cells.

Dependency of 5-azaC-induced DSBs on transcription suggests at least two potential mechanisms: (1) aberrant transcription followed by translation of protein(s) required for the formation of DSBs (e.g., nucleases) or (2) requirement of the physical process of transcription. To begin distinguishing these possibilities, protein translation was inhibited in HPV+ cells (UMSCC47) treated with 5-azaC. The translation inhibitor, cycloheximide, did not diminish DSBs caused by 5-azaC (Figure S4) suggesting that 5-azaC induced DSBs may be dependent on the physical process of transcription. To determine if DNA damage after 5-azaC occurred at sites of active transcription in HPV+ HNSCC cells were treated with the uridine analog EU to mark areas of transcription. After inhibition of RNA polymerase I to remove the background of ribosomal RNA transcription [41], EU-labeled control and 5-azaC treated cells were immunostained with EU and ɣH2AX to simultaneously identify sites of transcription and DNA damage, respectively (Figure 5C). As expected, 5-azaC treatment markedly induced ɣH2AX, and in these ɣH2AX-positive cells, EU and phosphorylated H2AX partially colocalized, while colocalization was not observed in untreated cells (Figure 5C,D). While ɣH2AX staining does mark DNA damage, we recognize that it is not equivalent to DSBs; however, colocalization of transcription and DNA damage combined with a loss of 5-azaC-induced DSBs in the absence of transcription is consistent with the possibility that sites of active transcription are sites of DSBs formation after 5-azaC treatment.

Since transcription is required for DSBs formation after 5-azaC treatment in HPV+ HNSCC, its inhibition may diminish toxicity of demethylation. To determine if blocking the formation of DSBs in HPV+ cells through transcriptional inhibition changes sensitivity to 5-azaC, clonogenic survival assays were performed using increasing doses of 5-azaC in the presence or absence of actinomycin D (Figure 5E). Although inhibition of transcription itself is toxic to cells, inhibition of transcription when added to 5-azaC treatment rescued a portion of colonies relative to cells treated with 5-azaC alone (Figure 5E). Taken together, these data suggest that 5-azaC induced DSBs in HPV+ HNSCC cells are dependent on active transcription and that transcription is required for a portion of the 5-azaC cytotoxic effect.

**Figure 6:**
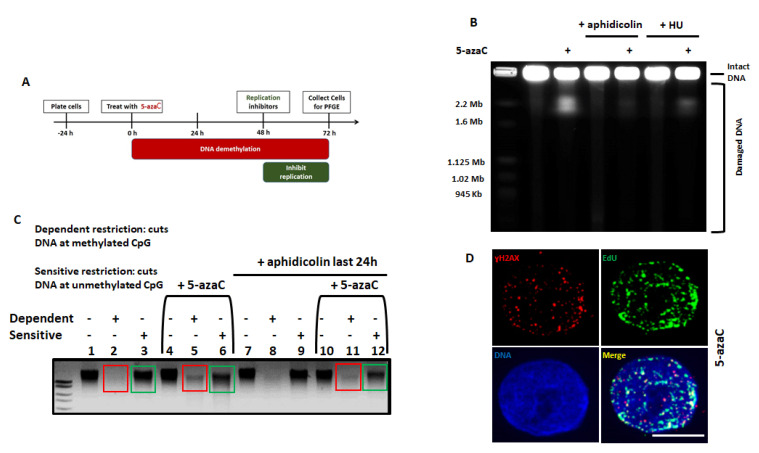
5-azaC induced DNA double strand breaks in HPV+ HNSCC depend on replication. (**A**) Schema of 72 h 5-azaC treatment with inhibition of replication during the last 24 h before analysis. (**B**) PFGE depicting HPV+ UMSCC47 cells with and without treatment with 20 µM 5-azaC for 72 h and treated or not with 3.3 µM aphidicolin or 1 mM hydroxyurea (HU) for the last 24 h before analysis as indicated. (**C**) DNA gel following dependent or sensitive restriction of DNA derived from UMSCC47 cells treated or not with 20 µM of 5-azaC for 72 h with addition of 3.3 µM aphidicolin in the last 24 h where indicated. (**D**) Coimmunostaining of EdU (active replication) and ɣH2AX in HPV-positive UMCC47 cells treated with 10 µM of 5-azaC for 72 h. Scale bar is 5 µm.

Cells have developed strategies to avoid or resolve collisions of transcriptional machinery with advancing replication forks because of the disastrous effects of collisions that result in genomic instability and double strand breaks formation [42,43,44,45]. Our data suggest that 5-azaC toxicity and DSBs depend on transcription (Figure 5B,E), and previous reports indicate that 5-azaC induces DNA damage dependent on active replication [20]. To determine whether the demethylation associated DSBs observed in HPV+ HNSCC also depend on replication, cells were treated with 5-azaC and then replication inhibitors (aphidicolin or hydroxyurea) in a schema similar to that used to explore the effect of transcription inhibition (Figure 6A). Prolonged exposure to hydroxyurea results in double strand breaks through collapse of replication forks [46], but here, short-interval hydroxyurea treatment (20 h) was employed as a replication inhibitor rather than as an inducer of DNA DSBs. Since azanucleosides must be incorporated into DNA during replication to inhibit DNA methyltransferases and cause demethylation [30], HPV+ UMSCC47 cells were treated for 48 h with 5-azaC to allow incorporation into DNA before addition of replication inhibitors (schema in Figure 6A). Both aphidicolin and HU diminished DSBs detection following 5-azaC treatment (Figure 6B). These results indicate that 5-azaC induced DNA double strand breaks in HPV+ HNSCC depend on both transcription and DNA replication.

Since 5-azaC must be incorporated into DNA to cause demethylation, replication inhibitors could impede 5-azaC-induced demethylation; therefore, DNA demethylation was confirmed in cells treated with replication inhibitors, using restriction enzymes specific to methylated CpG sites (“dependent restriction”) or unmethylated CpG sites (“sensitive restriction”; Figure 6C). In untreated cells, an intense uncut band was observed after sensitive restriction (green square, lane 3) but not after dependent restriction (red square, lane 2), showing that in the absence of 5-azaC treatment genomic DNA in HPV+ HNSCC is highly methylated. After treatment with 5-azaC, uncut DNA after sensitive restriction was diminished (lane 6 vs. 3) while uncut DNA after dependent restriction became detectable (lane 5 vs. 2). These data confirm that 72 h of 5-azaC treatment caused DNA demethylation. In 5-azaC treated cells exposed to aphidicolin during the last 24 h of treatment, a similar pattern was detected, confirming that treatment with replication inhibitors during the last 24 h of the experimental schema (Figure 6A) did not prevent DNA demethylation due to 5-azaC treatment (Figure 6C).

Finally, labeling of replication sites with thymidine analogue EdU during 5-azaC treatment and coimmunostaining of EdU with ɣH2AX showed a partial colocalization of replication sites with the DNA damage marker (Figure 6D).

### 2.4. 5-azaC-Induced DNA DSBs Are Randomly Distributed in the Genome but Are Enriched in Demethylated DNA

**Figure 7:**
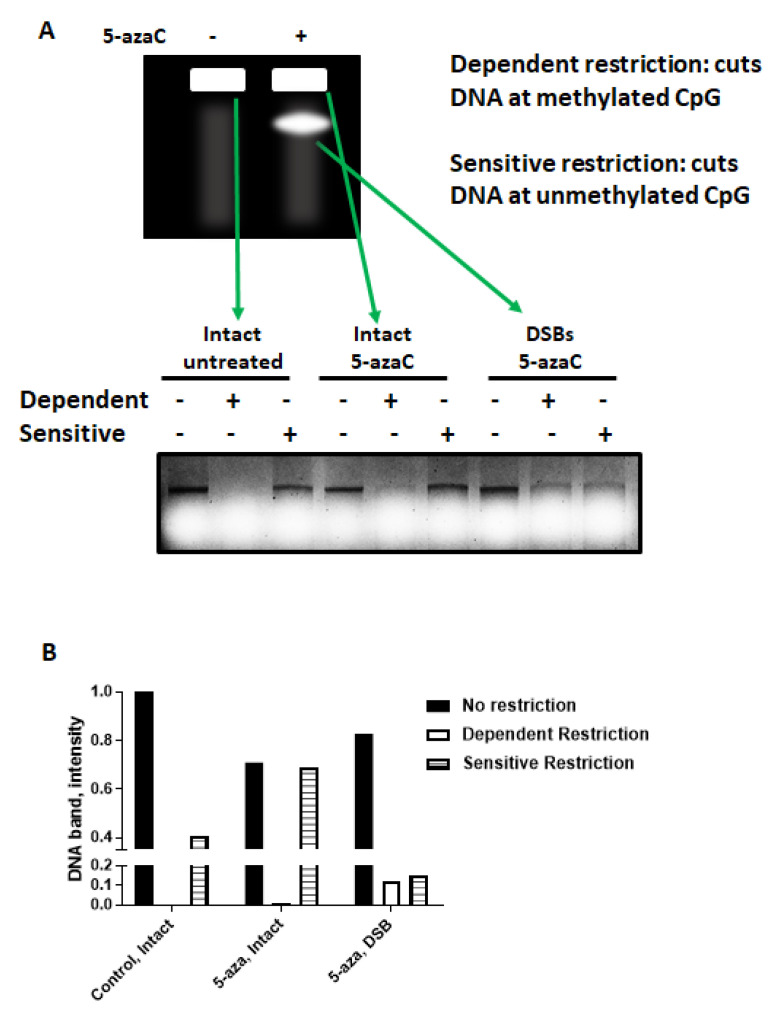
5-azaC induced DNA double strand breaks in HPV+ HNSCC are randomly distributed in the genome, but occur preferentially on demethylated DNA. (**A**) Schematic representation of PFGE (top) and DNA gel (bottom) showing results of sensitive and dependent restriction of DNA corresponding to control (untreated) and 5-azaC-treated (intact or DNA DSBs) purified from PFGE performed with HPV+ UMSCC47 cells. (**B**) Quantification of the DNA gel from (**A**).

To determine if demethylated DNA is associated with 5-azaC-induced DNA DSBs in HPV+ HNSCC, intact genomic DNA or DSBs from 5-azaC treated or control cells was gel purified following pulsed field gel electrophoresis then subjected to methylation dependent or sensitive restriction (Figure 7A). As expected, DNA from untreated cells was largely methylated with no band detectable after dependent restriction. After 5-azaC treatment, intact DNA had a faint band detected after dependent restriction, but a much more intense band after sensitive restriction suggesting that the majority of intact DNA after 5-azaC remained methylated (Figure 7A). In contrast, DNA isolated from the PFGE band containing DSBs demonstrated similar band intensities following dependent and sensitive restriction, suggesting that after 5-azaC treatment unmethylated DNA represented a much larger portion of DNA in DSBs than in intact DNA (Figure 7A,B).

To understand if DNA DSBs are enriched in specific genomic locations after 5-azaC treatment, whole genome sequencing of intact and damaged DNA isolated from 5-azaC-treated cells and purified from a pulsed field gel was analyzed. Initial analysis through alignment of reads to chromosomes revealed no marked differences between DNA from DSB versus intact DNA with 8% of the reads corresponding to exons and similar proportions mapping to intron, exons, or intergenic regions in both, intact DNA and double strand breaks, samples; no strand bias was found for the genes in either intact or DSB DNA. DNA from DSBs and intact DNA contained reads that mapped randomly to the genome with more reads mapping to longer chromosomes.

These results demonstrate that 5-azaC-induced DSBs in HPV+ HNSCC were enriched in demethylated DNA and randomly distributed in the genome.

### 2.5. Demethylation Alters the Protein Complement Associated with Chromatin in HPV+ HNSCC

To begin identifying pathways that may underlie the replication- and transcription-dependent DNA DSB formation observed in HPV+ HNSCC after 5-azaC, proteins associated with chromatin after demethylation were identified using tandem mass spectrometry. Demethylation increased the number of proteins associated with chromatin from 583 in untreated cells to 656 after 5-azaC (Figure S5) of these proteins, 499 were identified in both treated and untreated cells, 84 were observed only in untreated cells, and 157 were found only after 5-azaC treatment. Based on proteins relocated to chromatin after demethylation, gene ontology pathways were identified (Figure S5). The majority of significant pathways identified related to RNA splicing and processing, but also chromatin organization Interestingly, gene set enrichment analysis revealed that proteins relocating to the chromatin after demethylation are primarily associated with RNA metabolism and chromatin organization (Figure S5).

## 3. Discussion

DNA damaging drugs and radiation are a mainstay of cancer therapy, especially for patients with head and neck squamous cell carcinoma; however, damage to normal tissues from these therapies results in lifelong morbidity with related functional deficits with gastric-tube dependence in over 10% of patients receiving chemoradiation for late stage oropharyngeal cancer [47]. Acute toxicities from chemotherapy and radiation also limit the dose and effectiveness of these therapies. In addition to morbidity, increased non-cancer mortality is now being recognized in patients completing treatment with concomitant chemotherapy and radiation. Late cranial neuropathies have been described in up to 14% of patients with a mean latency of 7.7 years [48], and close to one quarter of patients treated with chemoradiotherapy will be admitted for aspiration pneumonia within 5 years of therapy and almost one-third of these patients will not survive this complication [49]. The high incidence of treatment related complications has accelerated the search for effective and less morbid therapy. Recent studies are exploring therapeutic de-escalation with the thought that decreasing dose or field of radiation will decrease morbidity [50]. Identification of patients with HPV+ OPSCC for deintensification of standard therapy will be an important advancement for decreasing morbidity and mortality. The cooperative group trial, E1308, used induction chemotherapy to select favorable patients for de-escalation, but with mixed results [51]. We identified genetic prognostic markers [9,52,53,54,55] and have used deep learning [56,57] and using circulating HPV DNA as alternative methods to identify high risk patients [58]. As opposed to deintensification of standard therapies, new treatment options that selectively target intrinsic susceptibilities are needed, especially for younger patients with HPV-associated HNSCC [5].

Given the intrinsic sensitivity of HPV+ HNSCC to 5-azaC treatment [21] and the tolerable side effect profile of this FDA-approved drug, 5-azaC emerges as an attractive therapy. Demethylating drugs are used for cancer therapy and have been associated with DNA damage; however, the cause and the type of DNA damage have not been well characterized [20,25]. We previously reported the effects of demethylating treatment to stabilize and activate p53 in HPV+ HNSCC. Here, we explored the type and effects of DNA damage induced by 5-azaC in HPV+ HNSCC to explore its contribution to 5-azaC cytotoxicity.

We found that 5-azaC and decitabine induced DNA double strand breaks in HPV+ HNSCC (Figure 2 and Figure 3), and confirmed and expanded reports that HPV+ cells are defective for homologous recombination (Figure 4B,C). Our studies showed that Rad51 did not colocalize to the sites of DNA damage in HPV+ HNSCC after HU treatment (Figure 4B) and that HPV+ cells did not activate a homologous recombination reporter after nuclease-induced DNA strand breaks (Figure 4C) [22,23]. Using BRCA2 deficient cells, we found that HR defects sensitize cells to demethylation (Figure 4A) consistent with reports that 5-azaC-induced DNA double strand breaks require homologous recombination [20]. Previously we reported that demethylation increased p53 levels and activity in HPV+ HNSCC resulting in cellular toxicity. Here, our data suggest that HR defects in HPV+ head and neck cancer cells also contribute to HPV+ HNSCC sensitivity to 5-azaC.

Since altered transcriptional activity is associated with DNA damage [33,59] and a consequence of demethylation is induction of global transcription [60,61], the requirement for transcription in 5-azaC-induced DNA DSBs was explored. We found that DSBs depended on active transcription (Figure 5) but did not depend on new protein synthesis (Figure S4) suggesting that the process of transcription, not a product of transcription, was mechanistically involved in DSBs formation. The dependence on active transcription also provides evidence against a model of 5-azaC induced DSBs involving replication fork collapse at DMNT1-DNA crosslinks as these lesions would form in the absence of transcription [62,63,64].

Transcription creates vulnerable DNA structures such as ssDNA exposed in the transcription bubble that have increased susceptibility to spontaneous deamination and mutagenic events [65,66]. Induction of aberrant or massive transcription also increases the chance of conflicts between replication and transcription machinery. Since 5-azaC toxicity depends on replication [20], the finding that replication was required for DNA DSB formation (Figure 6) in response to 5-azaC was not unexpected and is consistent with studies demonstrating that transcription-associated double strand breaks often involve conflict between the transcription and replication machinery [42,44,45,67,68,69,70]. In eukaryotic cells, these lesions generally arise from two general mechanistic etiologies: (1) through DNA–RNA hybrids that form from the hybridization of the newly transcribed mRNA with template DNA, thus causing an impediment to replisome progression [45,71,72], and (2) topological stress created by the anchoring of eukaryotic DNA to nuclear pore complexes at sites of transcription that does not allow the free rotation of DNA as the DNA and RNA polymerases traverse the DNA [45,70]. Both of these scenarios can result in replication fork collapse and the induction of transcription-associated recombination, leading to DSB formation [44,45]. We are beginning to explore if these potential mechanisms are involved in 5-azaC-induced DSBs in HPV+ HNSCC.

A question central to 5-azaC-induced DSBs in HPV+ HNSCC remained: what key molecular differences between HPV+ and HPV- cells allow the DSBs formation in HPV+ (Figure 2 and Figure 3), but not HPV- HNSCC cells (Figure S3) after demethylation therapy? We were exploring an additional mechanistic explanation, but one possibility is impaired homologous recombination repair found in several HPV-positive head and neck cancer cells (Figure 4 and [22,23]).

Demethylation is promising for therapy for HPV+ HNSCC since it reactivates p53 and increases apoptosis. As an additional mechanism of 5-azaC activity in HPV+ HNSCC, we described here that HPV+ HNSCC generate DNA DSBs from demethylation treatment and are at least partially deficient in repair pathways likely causing persistence of damaged DNA. We demonstrated here that these transcription and replication dependent DNA DSBs occur in both, cell lines and early passages of patient derived cells, and contributed to cellular toxicity; however, further studies are needed to determine if DSBs contribute to cytotoxicity HPV+ head and neck tumors after patients are treated with 5-azaC. The findings presented here serve as a model for further mechanistic understanding of induction of DNA double strand breaks in HPV+ HNSCC cells.

Previous studies demonstrated that many RNA-interacting proteins relocate to damaged chromatin [73], suggesting that they may be involved in DNA damage repair. Our analysis revealed that proteins associated with chromatin after 5-azaC treatment are enriched in RNA-binding and RNA processing proteins (Figure S5). A detailed functional analysis of these protein groups is being explored in our laboratories to increase understanding of the complex nature of transcription- and replication-dependent DNA DSBs produced by 5-azaC in HPV-positive head and neck cancer cells. A comprehensive mechanistic understanding may help to determine if subsets of HPV+ HNSCC tumors have different susceptibilities to 5-azaC toxicity through DSB induction and may suggest additional avenues to explore more effective therapeutic regimens.

## 4. Materials and Methods

### 4.1. Cells

HPV-negative (UNC7 and SCC61) and HPV+ (SCC090, UMSCC47, and UMSCC104) HNSCC cell lines were used. HPV- cells were cultured in DMEM/F12 medium supplemented with 0.4 μg/mL hydrocortizone, and HPV+ cell lines were grown in DMEM with nonessential amino acids. V-C8 and V-C8B2 cells were previously described [22]. All media was supplemented with 10% FBS (Thermo Fisher Scientific, Waltham, MA, USA), 50 μg/mL penicillin, and 50 μg/mL streptomycin (Thermo Fisher Scientific). All cell lines were tested negative for mycoplasma and authenticated by microsatellite testing.

To establish primary HNSCC cultures, surgical specimens were collected from consented patients in PBS within 30 min of resection. Tissue was cut into 5 mm^3^ pieces, disinfected by immersion in 70% ETOH for 1 min, rinsed with PBS four times, and digested in 0.05% trypsin-EDTA supplemented with collagenase type 1A (200 units/mL) (Sigma C-9722, St. Louis, MO, USA) in a vented flask at 37 °C with 5% CO_2_ for 10–20 min. Digestion was stopped by adding 1 volume of FBS (Sigma). After centrifugation at 1500 rpm × 5 min, the supernatant was aspirated, the cells were resuspended in keratinocyte serum-free medium with supplements and 10% FBS, strained through a 100 μm nylon cell strainer (Falcon; Becton Dickinson Labware), plated in keratinocyte serum-free medium with supplements (Gibco, Gaithersburg, MD, USA/Invitrogen, Waltham, MA, USA) and 10% FBS onto 0.1% gelatin (Millipore, St. Louis, MO, USA) coated plates, and grown at 370 C in a 5% CO_2_ incubator. The next day, cells were washed with PBS and grown in keratinocyte serum-free medium with supplements until they reached 90% confluence. After near confluence, the cells were detached with 0.05% trypsin-EDTA, the reaction was stopped with defined trypsin inhibitor (Gibco), and the cells were plated on uncoated plates in keratinocyte serum-free medium with supplements for experimental use.

### 4.2. Transfection

Cells were transfected using Lipofectamine 2000 (Invitrogen) according to manufacturer recommendations.

### 4.3. Drugs

5-Azacytidine, hydroxyurea, aphidicolin, triptolide, DRB, Actinomycin D, puromycin, and cycloheximide were obtained from Sigma.

### 4.4. Homologous Recombination Reporter

HPV-positive and negative cells were transfected with pDRGFP (a gift from Maria Jasin (Addgene plasmid # 26,475; http://n2t.net/addgene:26475; RRID:Addgene_26475) 24 and stable clones were selected on puromycin. Cells stably expressing pDRGFP were transfected with pCBASceI (a gift from Maria Jasin (Addgene plasmid # 26,477; http://n2t.net/addgene:26477; RRID:Addgene_26477) 25 and GFP positive cells were determined by FACS (BD FACSCalibur) 48 h after transfection

### 4.5. Immunoblotting

Immunoblotting was performed as previously described [25,26,27]. Briefly, cells were collected by trypsinization and lysed in radioimmunoprecipitation assay (RIPA) lysis buffer (Sigma) with the addition of protease inhibitors (Roche, Indianapolis, IN, USA) and phosphatase inhibitors (Sigma) for 15 min on ice. Insoluble material was removed by centrifugation at 14,000 rpm for 15 min at 4 °C. Proteins were separated in Tris-glycine polyacrylamide gels (Mini-PROTEAN; Bio-Rad, Hercules, CA, USA) and electrophoretically transferred onto polyvinylidene fluoride membranes. Membranes were blocked with 3% BSA in PBS and incubated with antibodies against ɣH2AX (Abcam, Cambridge, MA, USA), and tubulin (Santa Cruz, Dallas, TX, USA). After incubation with primary antibodies, membranes were washed, incubated with secondary DyLight antimouse and antirabbit antibodies (Thermo Scientific), and signals were visualized using a Bio-Rad imager. The uncropped immunoblotting image was shown in Figure S6.

### 4.6. Pulsed-Field Gel Electrophoresis

Cells were treated with indicated drugs, collected by trypsinization, resuspended in 1% InCert-agarose (in 37 °C PBS) to a final concentration of 1.5 million cells/100 μL, and agarose plugs were separated by pulsed-field gel electrophoresis as previously described [28].

### 4.7. Immunofluorescence

Cells were grown in chamber slides, treated, fixed, immunostained, and analyzed as previously described [25]. Cells with more than 10 foci were determined as positive. The primary antibodies used were mouse anti-ɣH2AX (Abcam) at a dilution of 1:2000 and rabbit anti-Rad51 (Santa Cruz) at a dilution of 1:500. Secondary antimouse Alexa 555 and antirabbit Alexa 488 were from Invitrogen and were used at a dilution 1:1000.

For EdU/EU localization with γH2AX, Click-iT EdU Imaging Kits (Invitrogen) or Click-iT™ RNA Alexa Fluor™ 594 Imaging Kit (Invitrogen) were used according to their instruction and ɣH2AX was costained after that.

### 4.8. Colony Formation Assay

Cells were plated into six well plates at a density of 500 cells/well. The next day, the cells were treated with indicated drugs. After 1 week, when colonies could be observed, colonies were fixed and stained with methylene blue in methanol (4 g/L). Colonies consisting of more than 30 cells were subsequently counted.

### 4.9. DNA Purification and Restriction

DNA was purified using DNeasy Blood and Tissue Kits (Qiagen, Germantown, MD, USA). Of DNA 0.5 µg was incubated with or without restrictases from EpiTect Methyl II DNA Restriction Kit (Qiagen) at 37 °C for 6 h, the enzymes were inactivated at 65 °C for 20 min following the digestion, the reactions were run in 1% agarose (Invitrogen) gel, and DNA was visualized with ethidium bromide (Bio-Rad).

### 4.10. Subcellular Fractionation

Subcellular Protein Fractionation Kit for Cultured Cells (Thermofisher Scientific) was used to isolate cytoplasmic, nuclear soluble, chromatin bound, and membrane proteins from the same cells according to the manufacturer’s suggested protocol.

### 4.11. Mass Spectrometry

Mass spectrometry was performed at the Yale Mass Spectrometry and Proteomics Core on the chromatin bound fraction, obtained as described above. Results were deemed significant at 95% confidence.

### 4.12. Next Generation Sequencing and Analysis

After PFGE, intact DNA and DSBs bands were cut of the gel and DNA was purified using Qiagen Gel Extraction kit. Sequencing with 5 kb reads was performed at Yale Genomics Facility using Pacific Biosciences. Corresponding to intact DNA intact 8082 good reads and 4431 good reads corresponding to DNA DSBs were chopped down to 100 bp in size. The number of chopped reads was 215K and 114K for intact and DSBs, respectively. Reads were mapped to GRCh38 (Genome Reference Consortium Human Build 38) with Bowtie2 resulting in 92% reads mapped for both DSBs and intact. There was no strand bias in the mapping; intact DNA: 99,807 top and 99,531 btm; DSBs: 52,735 top and 52,355 btm.

### 4.13. Comet Assay

A comet assay was performed using CometAssay^®^ Kit (2 × 20 well slides) from Trevigen (Gaithersburg, MD, USA) according to the manufacturer’s instructions.

### 4.14. GSEA

Gene set enrichment analysis was performed using Broad Institute software (https://www.gsea-msigdb.org/gsea/index.jsp) [29,30].

## 5. Conclusions

Here, we demonstrated that 5-azacytidine treatment induces replication and transcription-associated DNA double strand breaks in HPV-associated head and neck cancer cells. Blocking replication or transcription prevented formation of DNA double strand breaks and reduced sensitivity of HPV-positive head and neck cancer cells to 5-azacytidine. We also found that 5-azacytidine-induced DNA double strand breaks are randomly distributed in the genome but are enriched in demethylated DNA. Results presented here provide evidence for a novel application of demethylation treatment in HPV+ HNSCC and serve as a model for further mechanistic understanding of the selective induction of DNA double strand breaks in these cells.

## Data Availability

The data presented in this study are available on request from the corresponding author.

## Supplementary Materials

The following are available online at https://www.mdpi.com/2072-6694/13/1/21/s1, Figure S1: PFGE of HPV+ UMSCC104 cells and HPV+ tonsillar cancer cell culture following 72 h of treatment with 5-azaC; Figure S2: PFGE showing HPV+ cell line UMSCC47 after the treatment with 1 or 10 µM of 5-azaC daily for 3 days; Figure S3: PFGE showing HPV-negative cell lines after the treatment with 30 µM of 5-azaC or 2mM of HU; Figure S4: 5-azaC-induced DNA DSBs in HPV-positive HNSCC do not depend on protein synthesis (PFGE) showing HPV-positive UMSCC47 cells treated with 20 µM of 5-azaC for 72 h with addition of cycloheximide (CHX) where indicated in the last 12 h; Figure S5: (A) Schematic representation of the proteins located on chromatin in HPV+ UMSCC47 cells treated or not with 5-azaC. (B) Gene set enrichment analysis (GSEA) of proteins relocated to chromatin in HPV-positive cells after 5-azaC treatment; Figure S6. The uncropped immunoblotting image of Figure 1A.
