# Supplementary material for: Global Genome Demethylation Causes Transcription-Associated DNA Double Strand Breaks in HPV-Associated Head and Neck Cancer Cells
Source: Cancers (Basel). 2020 Dec 23;13(1):21. doi: 10.3390/cancers13010021 (PMC7793113; doi:10.3390/cancers13010021)
Supplement: Supplementary file 1 [file cancers-13-00021-s001.pdf]

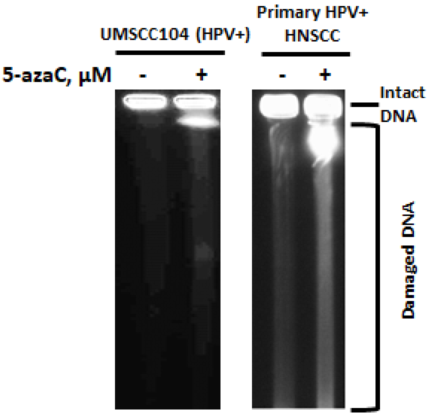

Figure S1. PFGE of HPV+ UMSCC104 cells and HPV+ tonsillar cancer cell culture following 72 h of treatment with 5-azaC.

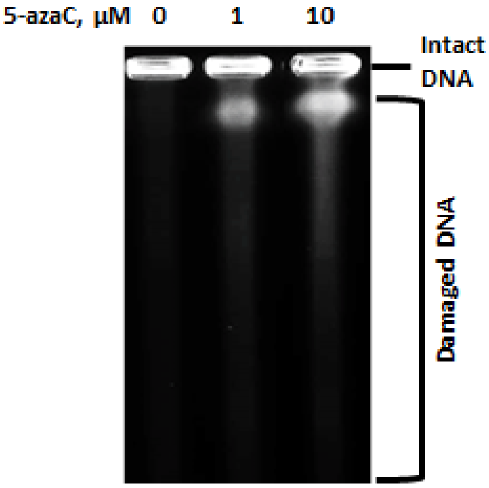

Figure S2. PFGE showing HPV+ cell line UMSCC47 after the treatment with 1 or 10 μM of 5-azaC daily for 3 days.

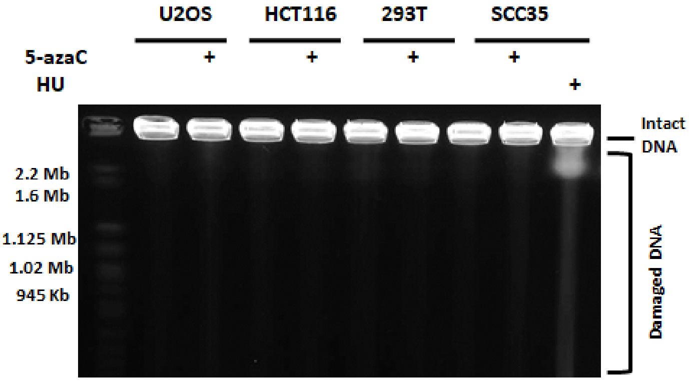

Figure S3. PFGE showing HPV-negative cell lines after the treatment with 30 μM of 5-azaC or 2mM of HU.

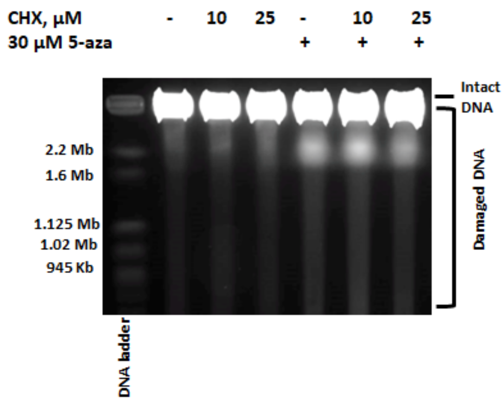

Figure S4. 5-azaC-induced DNA DSBs in HPV-positive HNSCC do not depend on protein synthesis (PFGE) showing HPV-positive UMSCC47 cells treated with 20 µM of 5-azaC for 72 h with addition of cycloheximide (CHX) where indicated in the last 12 h.

A

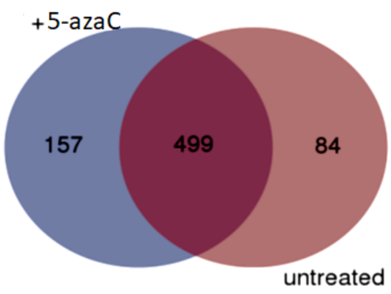

B

Gene Set Name [# Genes (K)]	Description	# Genes in Overlap (k)	k/K	p-value	FDR q-value
GO_RNA_PROCESSING [917]	Any process involved in the conversion of one or more primary RNA transcripts into one or more mature RNA molecules. [GOC:mah]	22		$4.7 e^{-19}$	$4.79 e^{-15}$
GO_MRNA_METABOLIC_PROCESS [840]	The chemical reactions and pathways involving mRNA, messenger RNA, which is responsible for carrying the coded genetic 'message', transcribed from DNA, to sites of protein assembly at the ribosomes. [ISBN:0198506732]	20		$2.91 e^{-17}$	$1.48 e^{-13}$
GO_RNA_BINDING [1901]	Interacting selectively and non-covalently with an RNA molecule or a portion thereof. [GOC:jl, GOC:mah]	26		$1.31 e^{-16}$	$4.45 e^{-13}$
GO_MRNA_PROCESSING [503]	Any process involved in the conversion of a primary mRNA transcript into one or more mature mRNA(s) prior to translation into polypeptide. [GOC:mah]	15		$1.74 e^{-14}$	$4.42 e^{-11}$
GO_RNA_SPLICING [433]	The process of removing sections of the primary RNA transcript to remove sequences not present in the mature form of the RNA and joining the remaining sections to form the mature form of the RNA. [GOC:krc, GOC:mah]	14		$4.74 e^{-14}$	$9.66 e^{-11}$
GO_RIBONUCLEOPROTEIN_COMPLEX_BIOGENESIESIS [419]	A cellular process that results in the biosynthesis of constituent macromolecules, assembly, and arrangement of constituent parts of a complex containing RNA and proteins. Includes the biosynthesis of the constituent RNA and protein molecules, and those macromolecular modifications that are involved in synthesis or assembly of the ribonucleoprotein complex. [GOC:isa_complete, GOC:mah]	13		$6.97 e^{-13}$	$1.18 e^{-9}$
GO_RNA_SPLICING_VIA_TRANSESTERIFICATIOTION_REACTIONS [345]	Splicing of RNA via a series of two transesterification reactions. [GOC:krc]	12		$1.52 e^{-12}$	$2.22 e^{-9}$
GO_CATALYTIC_COMPLEX [1355]	A protein complex which is capable of catalytic activity. [GOC:bhm, GOC:TermGenie, PMID:8077207]	19		$2.57 e^{-12}$	$3.27 e^{-9}$
GO_DNA_TEMPLATED_TRANSCRIPTION_ELONGATGATION [111]	The extension of an RNA molecule after transcription initiation and promoter clearance at a DNA-dependent RNA polymerase promoter by the addition of ribonucleotides catalyzed by an RNA polymerase. [GOC:mah, GOC:txnOH, PMID:15020047, PMID:18280161]	7		$1.44 e^{-9}$	$1.63 e^{-6}$
GO_CHROMATIN_ORGANIZATION [795]	Any process that results in the specification, formation or maintenance of the physical structure of eukaryotic chromatin. [GOC:mah, GOC:vw, PMID:20404130]	13		$1.86 e^{-9}$	$1.9 e^{-6}$

Figure S5. (**A**) Schematic representation of the proteins located on chromatin in HPV+ UMSCC47 cells treated or not with 5-azaC. (**B**) Gene set enrichment analysis (GSEA) of proteins relocated to chromatin in HPV-positive cells after 5-azaC treatment.

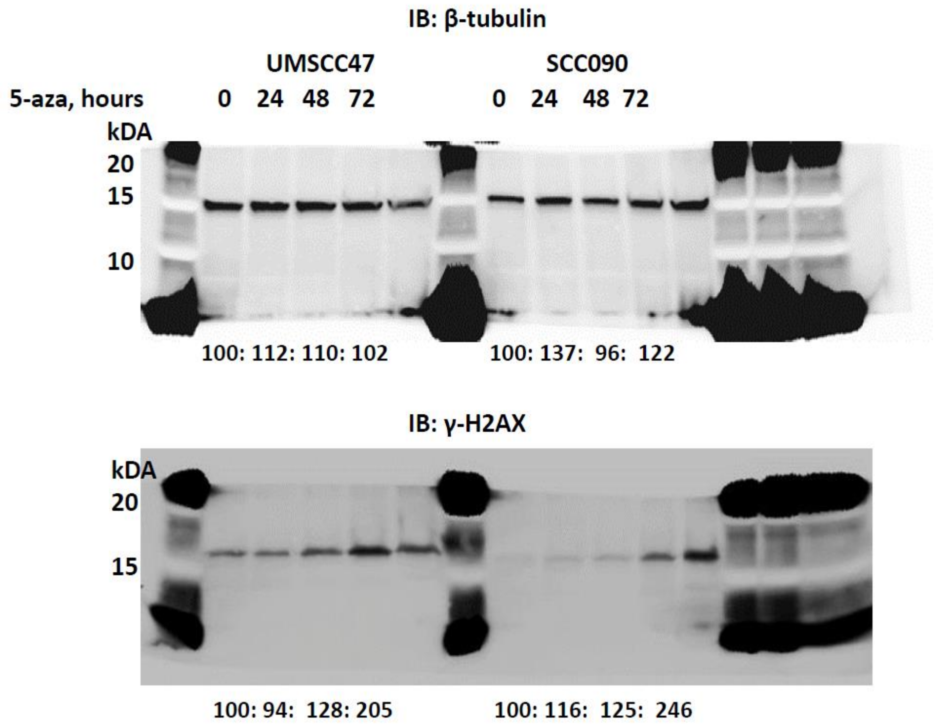

Figure S6. The uncropped immunoblotting image of Figure 1A.
